# Growth of arrays of oriented epitaxial platinum nanoparticles with controlled size and shape by natural colloidal lithography

**DOI:** 10.1186/1556-276X-9-336

**Published:** 2014-07-05

**Authors:** Vladimir Komanicky, Andi Barbour, Miroslava Lackova, Milena Zorko, Chenhui Zhu, Michael Pierce, Hoydoo You

**Affiliations:** 1 Faculty of Science, Safarik University, Park Angelinum 9, Kosice 04001, Slovakia; 2 Materials Science Division, Argonne National Laboratory, Lemont, IL 60439, USA; 3 National Institute of Chemistry, Hajdrihova 19, Ljubljana SI-1001, Slovenia; 4 Department of Physics, Rochester Institute of Technology, Rochester, NY 14623, USA

**Keywords:** Colloidal lithography, Platinum nanoparticles, Particle shape control

## Abstract

We developed a method for production of arrays of platinum nanocrystals of controlled size and shape using templates from ordered silica bead monolayers. Silica beads with nominal sizes of 150 and 450 nm were self-assembled into monolayers over strontium titanate single crystal substrates. The monolayers were used as shadow masks for platinum metal deposition on the substrate using the three-step evaporation technique. Produced arrays of epitaxial platinum islands were transformed into nanocrystals by annealing in a quartz tube in nitrogen flow. The shape of particles is determined by the substrate crystallography, while the size of the particles and their spacing are controlled by the size of the silica beads in the monolayer mask. As a proof of concept, arrays of platinum nanocrystals of cubooctahedral shape were prepared on (100) strontium titanate substrates. The nanocrystal arrays were characterized by atomic force microscopy, scanning electron microscopy, and synchrotron X-ray diffraction techniques.

## Background

Supported transition metal nanoparticles are widely used as catalysts and electrocatalysts in many industrial applications. Carbon-based electrically conducting supports are frequently used in the low-temperature proton exchange membrane fuel cells, while the refractory metal-oxide supports are used in moderate- and high-temperature applications such as automotive catalytic converters. Platinum is one of the most commonly used catalysts. Studies with single crystals [1] showed that catalyst activity can be influenced by the atomic arrangement of the catalyst surface as well as the presence of the defect sites. In the case of nanoparticulate catalysts, the shape can be an important governing factor in overall catalyst activity [2] because the nanoparticle shape is dictated by the crystallography of facets with the lowest surface energy. Each facet can have different specific catalytic activities. Particle-substrate interface crystallography and interfacial energy are an additional shape-controlling factor of supported catalysts [3]. The ability to fabricate well-defined model systems on various substrates where one can systematically vary the size, shape, and spacing between nanoparticles is of high fundamental [4] and practical importance [5]. Nanofabricated supported model catalyst systems can be probed with traditional scanning probe imaging techniques and synchrotron X-ray surface characterization tools. In the past, top-down nanofabrication techniques such as electron beam lithography (EBL) have been successfully used to produce platinum catalyst arrays [2,6,7]. Expensive instrumentation and multistep pattern transfer procedures make production of these systems challenging and costly. Additionally, EBL is a rather slow serial technique, and patterning of several square millimeters of the substrate area with densely packed arrays of dots can take many hours. For the practical applications, e.g., fuel cells, the total catalyst area has to be in the order of hundreds of square meters. There is clearly a motivation to produce well-defined catalyst samples supported on various substrates using cheaper and faster techniques. Natural lithography [8] alone or in combination with other techniques has been successfully used to produce metallic nanostructures and nanoparticle crystallites of random shape [9] and orientation [10]. The purpose of this report is to present a simple two-step process based on mask templates of a self-assembled silica colloidal sphere monolayer suitable for production of epitaxially oriented platinum nanoparticle arrays with precisely controlled shape. Shape and orientation of the particles are controlled via substrate crystallography, and particle size and spatial distribution are controlled via size of colloidal silica spheres used in monolayer template. We demonstrated only preparation of one type of particle shape, but it is possible to make different particle shapes if substrates with other crystallographic orientations are used [2,7]. Since the nanoparticles are supported on the annealable and electrically conducting Nb-doped strontium titanate (STO) substrates, the samples can be used both in electrocatalysis and gas phase catalysis.

## Methods

### Preparation of monodispersed colloidal silica spheres

Silica nanospheres were synthesized following the Stöber-Fink-Bohn method [11] starting from tetraethyl orthosilicate (TEOS 98%, Sigma-Aldrich, St. Louis, MO, USA), deionized water, ammonia (25%, Merck, Whitehouse Station, NJ, USA), and absolute ethanol (99.9%, Riedel-de Haën, Seelze, Germany) as precursor alkoxide, hydrolyzing agent, catalyst, and solvent, respectively. Two mother solutions were prepared: one containing ammonia-water and another one containing TEOS-ethanol. First, we add the ammonia-water solution to a solution of TEOS-ethanol kept at 50°C ± 1°C, in one step. Then, the solution was mixed and put back into the controlled water bath (50°C ± 1°C), for 1 h (no mixing). After 60 min, the resulting spheres were separated from the liquid phase with centrifugation and then ultrasonically dispersed in deionized water. The procedure was repeated three times. Then, the particles were dried in an oven at 50°C. Note that using this method, the final particle size critically depends on the reagent concentrations, molar ratio, and reaction temperature, so that difficulties are usually encountered in obtaining both a good control of the sphere size in a wide dimensional range and monodispersity with size distribution as narrow as possible. In this paper, we applied conditions for the synthesis of silica particles with well-defined particle size as described in [12]. We synthesized samples with nominal particle sizes of 150 and 450 nm.

### Preparation of monolayers of silica colloidal spheres on the STO substrates

The substrates are commercially available epi-polished (100)-oriented STO single crystals doped with Nb (MTI Corporation, Richmond, CA, USA; 0.7% to 1% Nb doping, resistivity 0.0035 to 0.007 Ω cm). The samples were etched for 4 min in a 3:1 mixture of concentrated nitric and hydrochloric acid, rinsed in deionized water, placed in a quartz tube, and annealed in air at 800°C; 0.2 wt.% of dried monodispersed colloidal silica was suspended in methanol using an ultrasonic bath. In order to deposit the monolayer of silica spheres, standard monodispersed colloidal spheres can be self-assembled into ordered 2D arrays using several approaches [13,14]. Initially, we used a method based on the transferring monolayer formed on the air-liquid interface by slowly draining colloid solution. This method works well for silica containing substrates such as glass slides. Investigation by atomic force microscopy (AFM) showed that this method used with STO substrates did not yield a continuous monolayer. Therefore, we directly micropipetted a colloidal silica sphere solution on the substrate squares with an area of 5 × 5 mm^2^. The solution contained enough silica spheres to give a full monolayer of colloidal silica spheres. A small droplet of water (approximately 10 μl) was also placed on top of the colloidal solution on the substrates. The solution on top of STO has been dried under continuous sonication. AFM images of deposited silica layers were acquired with a Bruker AFM model Icon (Bruker, the Netherlands). The silicone cantilevers were purchased from MikroMasch (Wetzlar, Germany) with a force constant of 14 N m^−1^. All images were acquired using tapping mode under ambient laboratory conditions. An epitaxial platinum film with a thickness of 8 nm was evaporated by e-beam evaporation using a three-step deposition technique [7]. A monolayer of silica beads was removed by sonication in hot concentrated potassium hydroxide aqueous solution. The nanocrystal arrays were characterized by X-ray diffraction (XRD) to confirm the orientation of crystalline platinum islands with respect to the substrate. The diffraction experiments were performed at the Advanced Photon Source (APS) using the four-circle diffractometer with a vertical scattering geometry at beamline 12BM. The incident energy was 11.5 keV, and beam defining slits were set to 1 mm with an under-focused beam. From our experience, intense synchrotron X-ray beam in the presence of oxygen from air causes damage to platinum single crystal surfaces. Most likely, this damage is a result of interaction between reactive free radicals generated from oxygen and platinum metal. We protected delicate nanocrystal arrays from X-ray damage by flowing ultra-high purity nitrogen gas into a polypropylene bag placed over the sample. For the STO (001) substrates, the Pt (004) and four (113) Bragg peaks were found. It is necessary to use a *θ*-offset of 0.15° to 0.30° for the *θ*-2*θ* scans so that the STO Bragg peak does not saturate the scintillation detector and to reduce background around the platinum Bragg peaks (STO and Pt (004) are separated by approximately 0.3° at 11.5 keV). The samples were also characterized by a high-resolution Hitachi Model S4700 scanning electron microscope (Hitachi, Tokyo, Japan) at the Electron Microscopy Center, Argonne National Laboratory.

## Results and discussion

### Microscopy characterization of silica monolayers and platinum nanoparticle arrays

**Figure 1:**
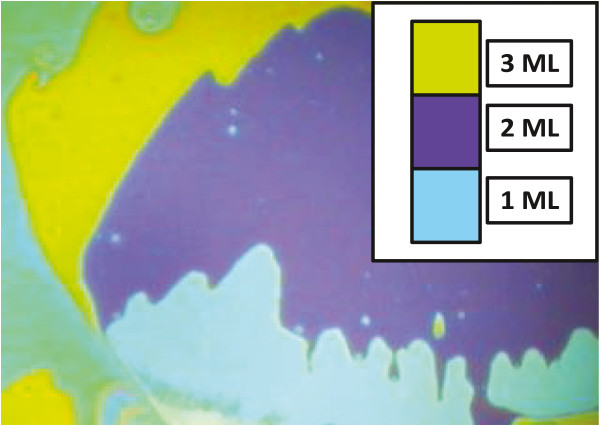
**Optical microscopy image of monolayer, bi-layer, and tri-layer made from 150-nm silica beads deposited on STO.** Light blue = monolayer, dark blue = bi-layer, and yellow = tri-layer.

Ordered silica bead monolayers, which later served as templates for the platinum metal deposition, were made by depositing solutions containing either 450- or 150-nm silica beads. We used AFM and optical microscopy to characterize deposited layers. Figure 1 shows optical microscopy image of 150-nm silica spheres deposited on STO. The colors indicate deposition of several monolayers when there are more silica particles than desired [15]; therefore, we decreased concentration of silica in the depositing solution until we obtain a single monolayer with a uniform color in the image. A similar procedure was performed with 450-nm beads. A single monolayer made from 150-nm silica has light blue color, as shown in Figure 1. This can be determined simply by finding a bare substrate below regions of the incompletely packed light blue layer. The number of layers can be verified by atomic force microscopy (AFM). Then, we optimized concentration of particles in the deposited solution until a single layer covered the majority of the substrate area.

**Figure 2:**
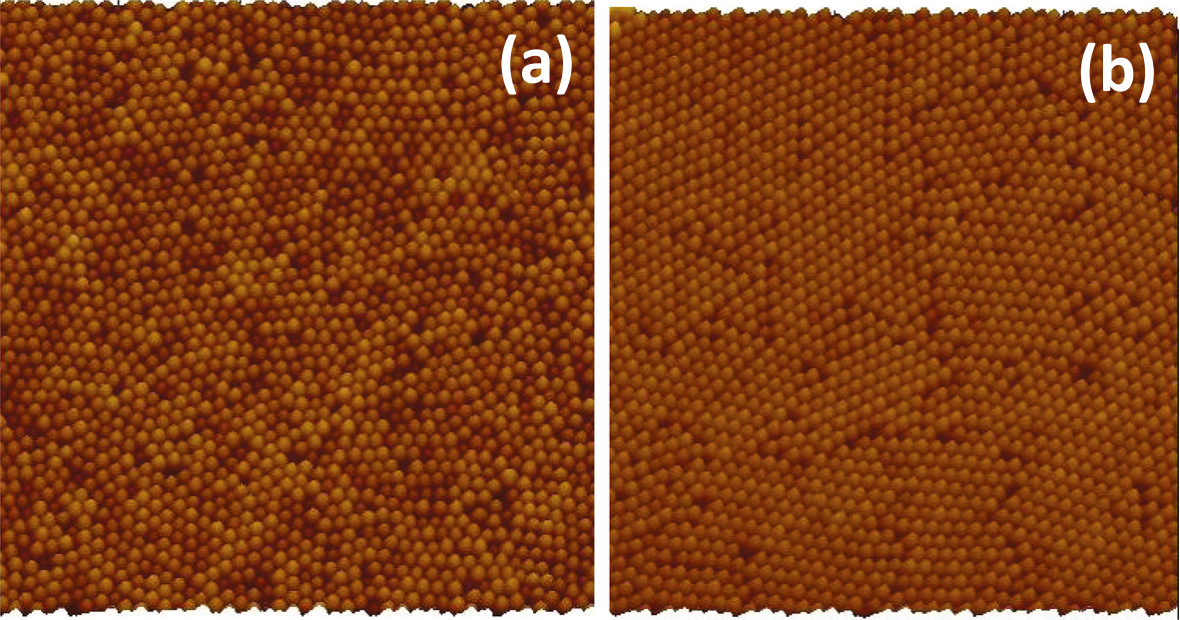
**AFM images of monolayers from silica beads with diameter (a) 150 nm and (b) 450 nm.** Imaged areas are 8 × 8 μm^2^ and 25 × 25 μm^2^, respectively.

**Figure 3:**
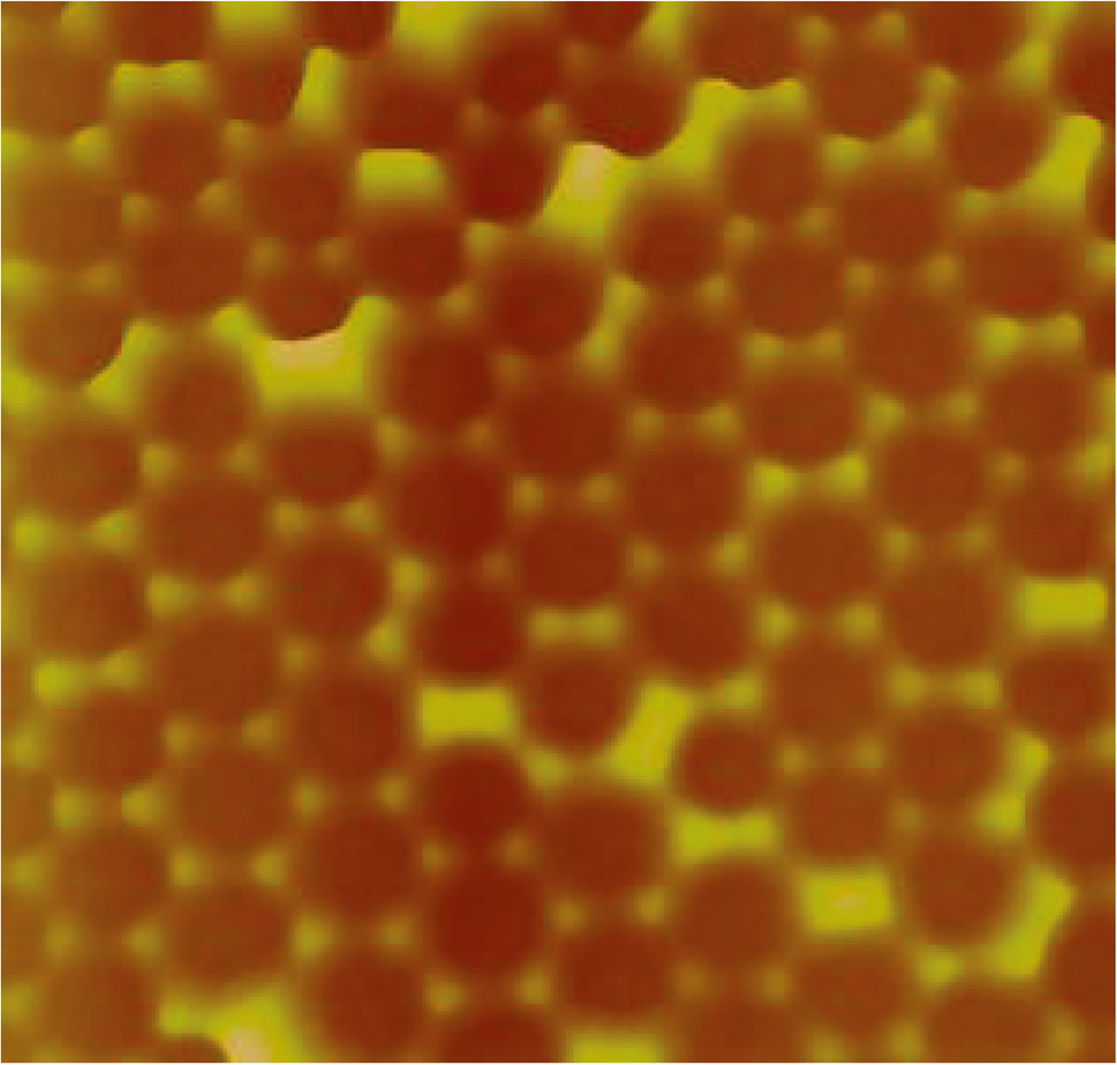
**AFM image of platinum nanoislands deposited through voids in template from hexagonally packed 450-nm silica beads.** Scanned area is equal to 3.5 × 3.5 μm^2^.

**Figure 4:**
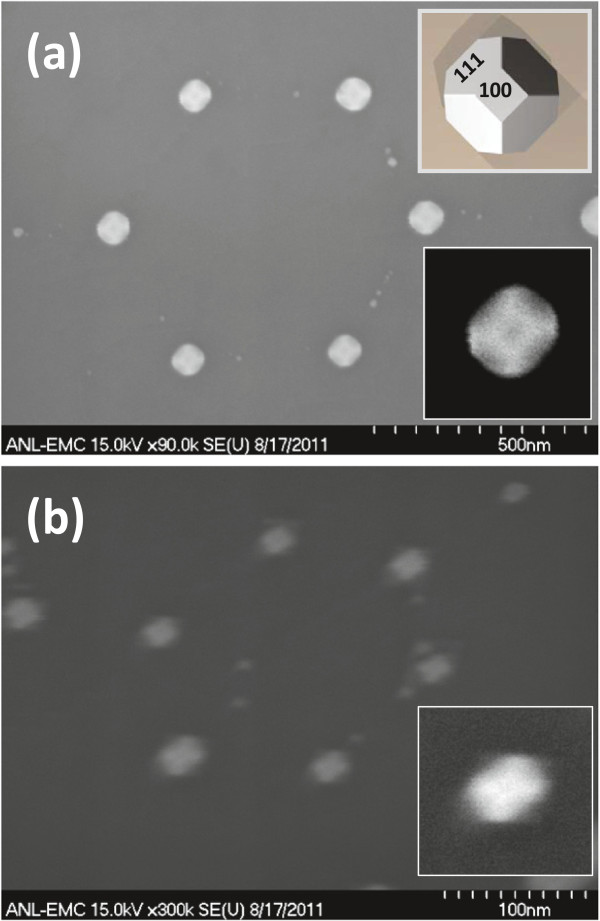
**SEM images of platinum nanocrystals.** The crystals are arranged in hexagonal patterns produced using 450-nm **(a)** and 150-nm **(b)** silica bead templates. Insets: top right corner, rendered particle; bottom right corners, digital zooms of actual cubooctahedral nanocrystals with clearly visible top 100 facets and four 111 facets on the sides. Distortion of hexagonal arrangement of nanocrystals in **(b)** is caused by the sample drift at high magnifications.

**Figure 5:**
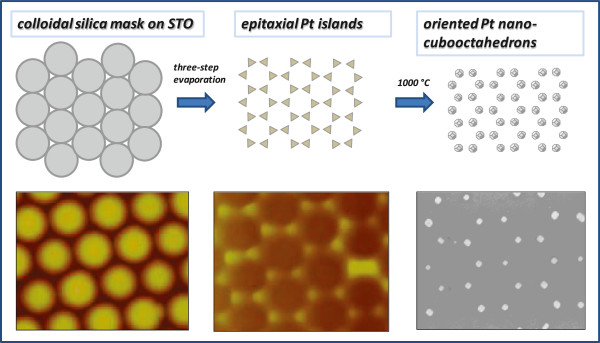
Schematic diagram summarizing production of arrays of platinum cubooctahedral nanoparticles on STO substrates.

Figure 2 shows AFM images of silica monolayers on STO prepared from 450- and 150-nm silica beads. Approximate particle count in both sample images is 1,800 particles. A common parameter used to characterize size distribution in nanoparticle batches is polydispersity index (PI). PI < 0.1 suggests a sample with high homogeneity in particle population [16]. The calculated PI for 150-nm particles is 0.055 and 0.023 for 450-nm beads. Both samples can be therefore considered monodisperse. Usual single domain size is several tens of particles for 150-nm silica beads; the domains made from 450-nm silica beads can contain several hundreds of particles. Because the monolayer deposition procedure was similar for both silica particle sizes, the higher uniformity of 450-nm silica beads leads to better monolayer crystallinity. It is possible that radial stress generated during drying of the colloid droplet [17] has some influence on the domain size, but we do not have much control over this parameter other than maintaining the drying time constant by keeping constant volume of colloid droplet in both cases. When colloidal spheres form two-dimensional, closely packed, hexagonal arrays on the STO substrate, a triangular void space exists among three neighbor spheres. These void spaces are arranged in hexagonal pattern. The void spaces serve as a physical mask through which we deposited platinum metal on the underlying STO substrate. The deposited material forms a hexagonal array of islands on the solid support. Each island has geometry of an equilateral triangle. One of the features of this technique is that the lateral dimension of the resulting Pt structures is much smaller than the diameter of the colloidal spheres. In order to deposit the epitaxial platinum layer, a three-step evaporation method [7] was used. During this process silica bead masks withstand temperatures close to 600°C without sintering and decomposition [18]. After metal deposition, a lift-off process was performed by removing the beads in hot concentrated solution of potassium hydroxide. Figure 3 shows AFM image of platinum islands deposited through triangular voids between hexagonally packed 450-nm silica beads. Imperfections in the bead packing result in formation of larger than expected platinum islands. The platinum islands were annealed in the furnace for 10 min at 1,000°C in nitrogen flow to protect them from oxidation. Cubooctahedral facetted particles form on (100) STO substrate [2]. Figure 4 shows SEM image of arrays of platinum nanoparticles prepared with 450- and 150-nm silica bead masks. The larger and smaller silica masks produced approximately 100-nm and approximately 20-nm platinum nanoparticles, respectively. The entire process is schematically shown in the Figure 5.

### X-ray characterization of Pt arrays on STO

**Figure 6:**
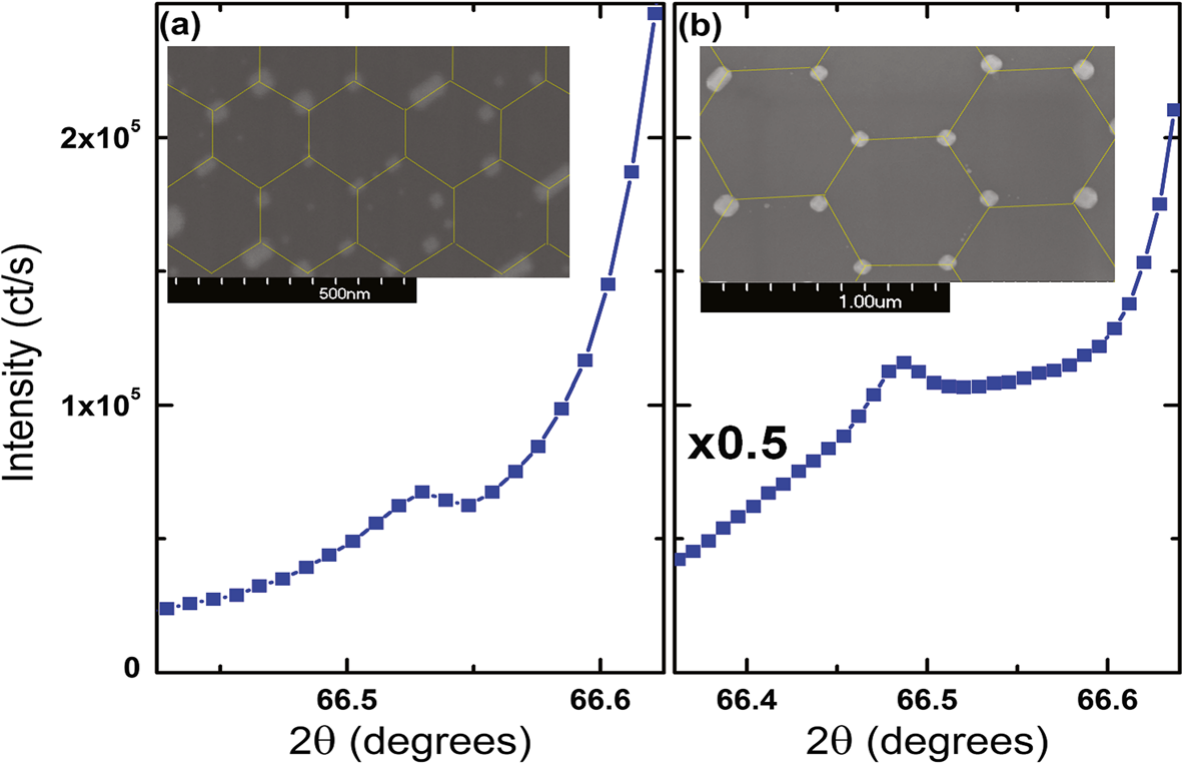
***θ*-2 *θ *scans.***θ*-2*θ* scans of Pt (004) for **(a)** 150-nm and **(b)** 450-nm samples showing that Pt (004) is parallel to the substrate's normal reflection. Insets show SEM images of the platinum particles after annealing (the hexagonal grids are guides to the eyes).

We performed X-ray diffraction (XRD) characterization of prepared nanoparticle arrays in order to prove the epitaxial relationship between particles and the STO substrate. The X-ray diffraction results for Pt nanoparticle arrays made using 150- and 450-nm silica bead templates are shown in Figure 6a,b, respectively. In both cases, there exists a Pt (004) reflection on the shoulder of specular STO (004); thus, the Pt nanocrystals have a surface normal to (001) facet, which agrees with Pt nanoparticles prepared by e-beam lithography [2] on STO (100). Because the peaks sit on the shoulder of strong reflection from STO, it is difficult to precisely estimate the width of the platinum peak.

**Figure 7:**
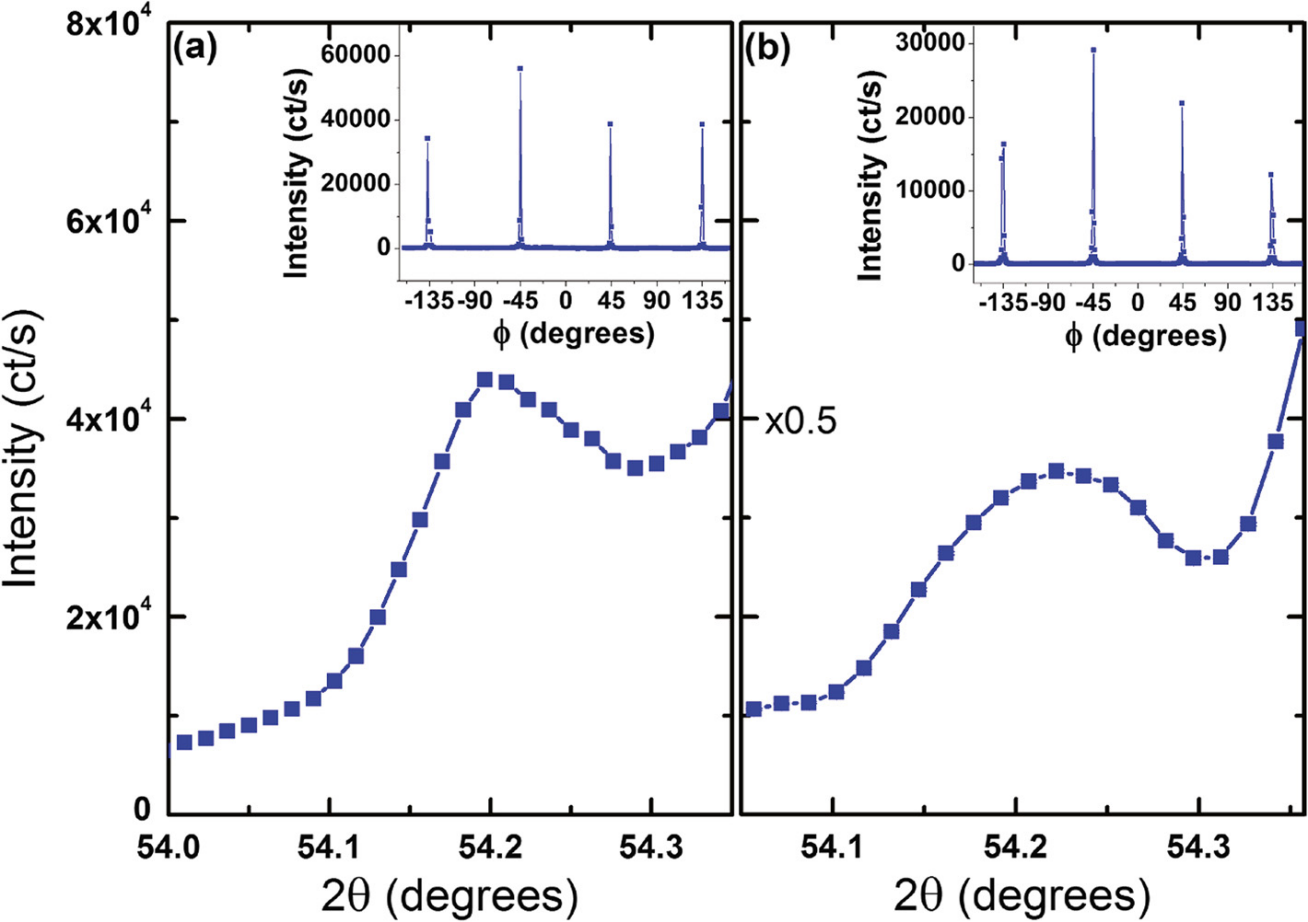
***ϕ*scans.***ϕ* scans of Pt (113) for **(a)** 150-nm and **(b)** 450-nm samples on the shoulder of STO (113) off-specular Bragg peak. Insets are the *H* = *K* = 1 (radius = √2 reciprocal lattice units) circle scans for *L* = 3 showing that Pt in-plane ordering is equivalent to STO as all peaks are separated by 90°. STO (200) is aligned to the direction of *ϕ* = 0.

In order to show in-plane epitaxial orientation of Pt nanoparticles, we performed scans in the *HK* directions. Figure 7 shows Pt (113) peak on the shoulder of the STO (113). The *ϕ* scans (constant *L*) shown in the insets of Figure 7a,b show that equivalent Pt (113) peaks occur every 90°, as expected, and no other Pt peaks are found in the *ϕ* scans. Figures 6 and 7 together show that the Pt nanocrystals are indeed epitaxially deposited onto the STO substrate.

## Conclusions

We have demonstrated a simple method for the preparation of platinum nanoparticle arrays with control of nanoparticle size, spacing, and shape. This method can be used to produce monodisperse platinum catalyst nanoparticles without need for elaborate nanopatterning equipment. Particle size and spacing can be controlled by the size of the silica beads used to form the monolayer template. The silica monolayers deposited at optimized conditions on Nb-doped STO were used as masks for deposition of epitaxial platinum islands. Because of initial epitaxial relation between platinum and STO, and annealing conditions, cubooctahedral platinum nanoparticles form. The platinum nanocrystal arrays were characterized by scanning electron microscopy and synchrotron X-ray scattering indicating that they are single crystalline and oriented. Because the STO substrate is electrochemically inactive in a very wide range of potentials in aqueous electrolytes, platinum nanoparticle arrays can be used as well-defined model electrocatalysts to study technologically important reactions such as oxygen reduction reaction, oxygen and hydrogen evolution reaction, or carbon monoxide oxidation. These reactions are important in operations of fuel cells and electrolyzers where platinum metal is the main constituent of deployed catalysts.

## Competing interests

The authors declare that they have no competing interests.

## Authors' contributions

VK designed the study, carried out the experiments, provided theoretical and experimental guidance, and drafted the manuscript. AB performed the XRD experiments and helped to draft the manuscript. ML performed the statistical analysis, carried out experiments, measured AFM images, and helped to draft the manuscript. MZ prepared silica particles. CZ helped with synchrotron scattering experiments. MP gave his help in using and maintaining the e-beam evaporator. HY provided theoretical and experimental guidance and helped to draft the manuscript. All authors read and approved the final manuscript.
